# Complementary Effect of Non-Persistent Silver Nano-Architectures and Chlorhexidine on Infected Wound Healing

**DOI:** 10.3390/biomedicines9091215

**Published:** 2021-09-14

**Authors:** Mykola Pernakov, Maria Laura Ermini, Oksana Sulaieva, Domenico Cassano, Marco Santucci, Yevhenia Husak, Viktoriia Korniienko, Giulia Giannone, Aziza Yusupova, Iryna Liubchak, Maria Teodora Hristova, Anton Savchenko, Viktoriia Holubnycha, Valerio Voliani, Maksym Pogorielov

**Affiliations:** 1 Sumy State University, 40007 Sumy, Ukraine; m.pernakov@med.sumdu.edu.ua (M.P.); e.gusak@med.sumdu.edu.ua (Y.H.); v.kornienko@med.sumdu.edu.ua (V.K.); ms.aziza.yusupova@gmail.com (A.Y.); irinalybchak@gmail.com (I.L.); sava20077@ukr.net (A.S.); v.golubnichaya@med.sumdu.edu.ua (V.H.); 2 Center for Nanotechnology Innovation @NEST, Istituto Italiano di Tecnologia, 56127 Pisa, Italy; laura.ermini@iit.it (M.L.E.); domenico.cassano@sns.it (D.C.); m.santucci14@studenti.unipi.it (M.S.); giulia.giannone@sns.it (G.G.); valerio.voliani@iit.it (V.V.); 3 Medical Laboratory CSD, 03148 Kyiv, Ukraine; oksana.sulaieva@gmail.com; 4 NEST-Scuola Normale Superiore, 56127 Pisa, Italy; 5 The University of Western Ontario, London, ON N6A 3K7, Canada; 6 Medical University of Pleven, 5800 Pleven, Bulgaria; mthristova79@gmail.com; 7 NanoPrime, 39200 Debica, Poland

**Keywords:** antimicrobials, surgical site infections, silver, nanomaterials, wound healing

## Abstract

Surgical site infection (SSI) substantially contributes each year to patients’ morbidity and mortality, accounting for about 15% of all nosocomial infections. SSI drastically increases the rehab stint and expenses while jeopardizing health outcomes. Besides prevention, the treatment regime relies on an adequate antibiotic therapy. On the other hand, resistant bacterial strains have currently reached up to 34.3% of the total infections, and this percentage grows annually, reducing the efficacy of the common treatment schemes. Thus, new antibacterial strategies are urgently demanded. Here, we demonstrated in rats the effectiveness of non-persistent silver nano-architectures (AgNAs) in infected wound healing together with their synergistic action in combination with chlorhexidine. Besides the in vivo efficacy evaluation, we performed analysis of the bacteriological profile of purulent wound, histological evaluations, and macrophages polarization quantifications to further validate our findings and elucidate the possible mechanisms of AgNAs action on wound healing. These findings open the way for the composition of robust multifunctional nanoplatforms for the translation of safe and efficient topical treatments of SSI.

## 1. Introduction

Surgical site infection (SSI) remains a common hospital-acquired infection and requires an adequate treatment strategy [1]. SSI occurs in 1.5–20% of surgical cases, depending on procedure type, country, and classification of wounds, significantly contributing to the costs of surgical procedures (up to EUR 19.1 billion annually in EU) [2]. *Staphylococcus aureus*, *Escherichia coli*, and *Pseudomonas aeruginosa* are the most usual bacteria associated with SSI, and the treatment regime relies on an adequate antibiotic therapy defined by the location and nature of the infection [3]. Despite the effective antibacterial treatment schemes, the number of resistant bacterial strains grows annually and has currently reached up to 34.3% of the total infections [4]. Infections caused by multidrug-resistant (MDR) microorganisms are associated with increased risks of complications and increased healthcare costs. Thus, the development of new antibacterial strategies is urgently demanded. In this regard, the employment of nanotechnology, especially of nanomaterials with antibacterial properties, currently represents the most promising approach to overcome microbial drug resistance [5,6]. In particular, some nanoparticles exhibit multiple mechanisms of action that affect microbial wall, organelles, and biochemical pathways [7]. Some organic and inorganic nanoparticles have been effectively applied for prevention and treatment of bacterial infections. Organic nanoparticles, such as an andrographolide and quaternized chitosan, exhibited significantly enhanced anti-infection and regeneration capabilities [8,9]. Metal-containing nanoparticles have been extensively investigated for the development of antimicrobial drugs, and silver nanoparticles resulted as one of the more promising [10]. Even though already employed more than 100 years ago, silver nanoparticles are only recently being touted as the last line of defense to reduce both the bacterial burden and the inflammatory response [11,12]. Indeed, silver nanoparticles can penetrate the bacterial cell wall and disrupt the signaling cascades essential for bacterial survival and colony expansion [11,13]. Although effective, noble metal-based treatments are precluded from clinics for the concern of metal persistence [14]. Indeed, noble metal nanoparticles are not (bio)degradable and their size usually leads to severe clearance issues with accumulation and persistence in excretory organs. In this regard, silver nano-architectures (AgNAs) are biodegradable nanoplatforms that may bring inorganic nanomaterials back to the forefront of clinical applications [15]. AgNAs are intrinsically sterile nanocapsules composed of 100 nm hollow silica nanospheres embedding ultrasmall silver nanoparticles in a polymeric functional matrix [16,17]. The silica shell both protects the material in the inner cavity from the environment and provides an easily modifiable/functionalizable surface [18]. Closely packed silver nanoparticles confer the chemo-physical behavior needed for therapeutic applications, while the polymer can be functionalized with active molecules of interest, such as drugs and peptides [19]. AgNAs are (bio)degradable, and their building blocks are excreted from organisms, addressing the long-standing challenge of metal persistence that hampers the clinical translation of noble metal nanotherapeutics [15]. Noticeably, the remarkable biosafety profile of AgNAs has been recently confirmed in whole-body toxicity experiments performed on vertebrate models [15].

Here, we demonstrated in rats the effectiveness of non-persistent silver nano-architectures for the treatment of infected wounds together with their synergistic action in combination with chlorhexidine (Ch). These findings are a significant advance in the translation of efficient topical treatments of SSI.

## 2. Materials and Methods

### 2.1. Materials

All chemicals were purchased from Sigma-Aldrich (Darmstadt, Germany) and used as received. Chlorhexidine (Ch) solution was purchased from Kilaff, Ukraine. Bacteria (*S. aureus*, *E. coli*, and *P. aeruginosa)* and U2OS cells were used from the Collections of Sumy State University. Bacteria culture media were received from HiMedia (Maharashtra, India). All reagents and media for cell culture experiments were from Gibco^®^ (Gaithersburg, MD, USA) and used as received.

The animal experiment was approved by the Commission on Bioethics Compliance in Experimental and Clinical Research of Sumy State University and carried out in accordance with the Directive 2010/63/EU of the European Parliament and of the Council of 22 September 2010 on the Protection of Animals Used for Scientific Purposes.

### 2.2. Material Synthesis

#### 2.2.1. Silver Nanoarchitectures

The following protocol was standardized for the production of 1.5 mg AgNAs in about 4 h. The protocol can be scaled-up to 20 mg.

##### Synthesis of Silver Clusters

Silver clusters with a size of about 1 nm were prepared according to the following procedure. A volume of 200 μL of reduced glutathione (100 mM) and 200 μL of AgNO_3_ aqueous solution (25 mM) were added to 20 mL of milliQ water, immersed in an ice bath. During vigorous stirring, 200 μL of sodium borohydride (8 mg mL^−1^ in milliQ water) were added quickly, and the mixture was vigorously stirred for 30 min. After the addition of NaBH_4_, the solution underwent some color changes until it became brilliant red. After removing the ice bath, 10 μL of poly(sodium 4-styrene sulfonate) (70 kDa, 30% aqueous solution) was added. The solution was stirred 15 min at room temperature.

##### Synthesis of Silver Nanoparticles Arrays 

A volume of 75 μL water solution of poly(l-lysine) hydrobromide, 15–30 kDa (PL, 40 mg mL^−1^), was added to 20 mL of the previously prepared silver cluster solution, and the mixture was gently stirred for 20 min at room temperature. The as-synthesized silver aggregates were collected by centrifugation (13,400 rpm for 3 min), suspended in 4 mL of milliQ water and sonicated for a maximum of 4 min.

##### Synthesis of AgNAs

A volume of 70 mL of absolute ethanol followed by 30 μL of tetraethyl orthosilicate (TEOS, 98%) were added in two 50 mL plastic Falcon tubes. The solution was stirred for 1 min at room temperature (RT). Of the silver nanoparticles arrays previously prepared, 4 mL were added to the Falcon tubes (2 mL to each), then, after 10 min, 70.5 μL of dimethylamine (40% stock, 428 mg/mL) was added to each tube, and the solution was gently shaken for further 3 h. The as-synthesized NAs were collected by 30 min centrifugation at 4000 rpm, washed twice with ethanol to remove unreacted precursors, and suspended in 1 mL of ethanol. A short spin centrifugation was employed in order to separate the structures of over 200 nm from the supernatant, which was recovered as a red iridescent solution. AgNAs remain usually stable for at least 1 year in EtOH. Product recovery: (i) 3 min centrifugation at 14,000 rpm, (ii) removal of the colorless supernatant, and (iii) addition of the solvent of interest. The solubility of NAs in water, buffers, and physiological fluids was tested for up to 60 mg mL^−1^.

#### 2.2.2. Nanoarchitecture Characterization

##### Transmission Electron Microscopy (TEM)

TEM observations of nanoparticles were carried out on a ZEISS Libra 120 TEM (Carl Zeiss, Oberkochen, Germany) operating at an accelerating voltage of 120 kV, equipped with an in-column omega filter. The colloidal solutions were deposited on 300 mesh carbon-coated copper grids and observed after at least 5 h.

##### Inductively Coupled Plasma Mass Spectrometry (ICP-MS) Analyses

Nanoparticles were dissolved in 1 mL ICP-MS grade HNO_3_ and digested by microwave irradiation (CEM, Matthews, NC, USA) at 200 °C for 15 min in borosilicate glass vessels. The resulting solution was diluted to 10 mL with ICP-MS grade water, and its silver content was determined by ICP-MS Agilent 7700 (Agilent Technologies, Santa Clara, CA, USA) analysis against a standard calibration curve.

#### 2.2.3. Antibacterial Activity Assessment

##### Minimum Inhibitory Concentration (MIC)

AgNAs antibacterial activity against *S. aureus, E. coli,* and *P. aeruginosa* was assessed via determination of the minimum inhibitory concentration (MIC), using the tube serial dilution method according to the International Recommendations provided by the Clinical and Laboratory Standards Institute (CLSI).

Prior to the test, microbial suspensions in Muller–Hinton broth were prepared at a concentration of 1.5 × 10^8^ CFU/mL from an overnight culture. Then, a serial dilution of the suspensions was performed to reach the final bacterial concentration of 1.5 × 10^6^ CFU/mL. Serial dilutions of Ag nano-architecture in Muller–Hinton broth were added to bacterial suspensions to get final concentrations of the silver ranging from 0.62 to 40.0 µg/mL and the final concentration of bacteria was 1.5 × 10^5^ CFU/mL. The tubes with medium and bacteria were used as a positive control. After all tubes were incubated 24 h at 37 °C, the AgNAs concentrations in the tubes with the lowest concentration of the investigated samples that completely inhibited visual growth of bacteria (no turbidity) were considered as the MIC.

##### Time-Dependent Inhibition Test

The *S. aureus*, *E. coli*, and *P. aeruginosa* strains, obtained from the National Collection of Microorganisms (D.K. Zabolotny Institute of Microbiology and Virology, Kyiv, Ukraine), were cultivated on nutrient agar for 24 h. Suspensions of their overnight cultures were mixed with nutrient broth, and the final concentration of microorganisms was adjusted to 10^6^ CFU using a McFarland standard.

A time-dependent inhibition test was performed using the broth microdilution method. Initially, a serial dilution of the silver nanoparticles was prepared in Muller–Hinton broth, and the concentration of silver varied from 5 to 20 µg/mL. Then, 20 µL of bacteria were added to each well with the final concentration of 10^5^ CFU/mL. The total volume of the tested sample was equal to 200 µL. Tubes containing tested samples in growth medium without bacterial inoculums and bacterial suspensions in the initial concentration were used as negative and positive controls, respectively. All tubes were incubated aerobically at 37 °C. In 2, 4, 6, 8, 12, 24, and 36 h, 10 µL of media from the wells was pipetted out and spread on an agar plate surface using a sterile metal spreader. The plates were incubated at 37 °C for 24 h. The colony-forming units (CFU) values were calculated by counting the visible colonies and then displayed in logarithmic form. All the experiments were triplicated.

##### Cell Toxicity Experiment

U2OS cells were used for determining the toxicity AgNAs in the resazurin reduction assay. The cells were cultured in Dulbecco’s modified Eagle’s medium/Nutrient Mixture F-12 (DMEM, Gibco, Gaithersburg, MD, USA) supplemented with 10% fetal bovine serum, 100 units/mL penicillin, 100 μg/mL streptomycin, and 2.5 μg/mL amphotericin B (Gibco, Gaithersburg, MD, USA) under the conditions of 37 °C and 5% CO_2_. The medium was replaced every 2 days. Cells were seeded to 96-well plates in a density of 5 × 10^3^ cells/well, and after 12 h incubation, AgNAs solution was added to the cells in concentrations of 5, 10, 20, and 40 µg/mL. Cell toxicities were assessed by an Alamar Blue colorimetric assay in 24 h, 2 and 3 days in repetition. Alamar Blue was added to each well in an amount equal to 10% of the medium volume. As a negative control, the Alamar Blue solution was added to the wells containing only culture medium without cells. As a positive control, Alamar Blue solution was added to the wells containing only cells without samples (TCP control). The plates were incubated for 4 h at 37 °C in the dark. 100 μL medium from each well was transferred to another 96-well plate, and the absorbance was measured using a Multiskan FC Microplate Photometer (Thermo Fisher Scientific, Waltham, MA, USA) at wavelengths of 570 and 600 nm. Cell visualization was provided using EVOS XL Core cell imaging system (Thermo Fisher Scientific, Waltham, MA, USA) on days 1, 2, and 3.

##### Animal Experiment Design

All procedures with laboratory animals were carried out under intravenous anesthesia (ketamine, 10 mg per 1 kg). Before the experiment, the surgical field (interscapular region) was shaven, treated with 70% ethanol, and covered with a sterile cloth. A rectangular wound defect up to subcutaneous layer sized 1.0 cm × 1.5 cm (S = 1.5 cm^2^) was excised using a sharp scalpel. A gauze swab with a mixture of the microorganisms *S. aureus*, *E. coli*, and *P. aeruginosa* (5 × 10^9^ CFU/mL each) was put into the wound and sewn up. The wound was open in 72 h after purulent inflammation occurrence, and the swab was removed with the remnant of pus. To realize different wound treatment strategies, animals were randomly allocated into three groups:

1. Control (24 rats)—application of 0.05% chlorhexidine solution;
2. “AgNAs” (24 rats)—application of 40 μg/mL solution of AgNAs;
3. “Chlorhexidine–AgNAs” (24 rats)—combined application of 40 μg/mL solution of AgNAs in 0.05% chlorhexidine.

After wound treatment procedures, sterile dressing was applied to prevent cross-contamination from the skin surface. The daily dressing was performed under aseptic conditions following the basic principles of purulent surgery.

The daily monitoring of wound defect with digital photography was carried out to evaluate wound size reduction rate. The area of the wound defects was calculated using the open-access ImageJ software.

Animals were humanly euthanized by an anesthesia overdose (ketamine, 70 mg/kg of animal weight) 3, 7, 14, and 21 days after treatment (6 animals in each time point). The skin samples were taken for further histological assessment.

##### Wound Microbiology

Detailed bacteriological analysis of the infected wounds was carried out by swabbing of the wound bed in the fixed period of the healing process. Specimen were collected on days 1, 2, 3, 5, 7, 9, and 11 after purulent wound modeling. All samples were examined using a classical bacteriological technique with primary inoculation on selective media by streak plate technique. Then, all Petri dishes were incubated at 37 ± 1 °C for 24 h. It was followed by determination of the number of the *E. coli*, *S. aureus*, and *P. aeruginosa* in the wound samples by colonies counting expressed in logarithmic form.

##### Histological Analysis and Immunohistochemical Markers Assessment

In addition to the observation of macroscopical features, the wound process was analyzed histologically. For this aim, tissue samples obtained after excision were fixed in 10% neutral buffered formalin and processed to paraffin-embedded blocks that were cut at 4 μm thickness and stained both with hematoxylin and eosin (HE) and immunohistochemically (IHC), according to standard procedures and protocols.

The histological evaluation covered the basic phases of the wound healing process including inflammation, proliferation associated with granulation tissue formation, angiogenesis and epithelization, and, finally, remodeling. The inflammatory reaction was evaluated semi-quantitatively by assessing the density of acute inflammatory infiltration (1—mild, 2—moderate, and 3—severe infiltration) by polymorphonuclear leukocytes (PMN). The same approach was applied when estimating granulation tissue formation. Angiogenesis was analyzed using IHC staining with antibodies against CD31 (DAKO, Clone JC70A) and VEGF (DAKO, Clone VG1). Cell proliferation was evaluated by counting Ki-67 positive cells (DAKO, Clone MIB-1).

As macrophages are considered as key regulators of the wound process, switching inflammation to proliferation, the IHC study using antibodies against CD68 (DAKO, Clone KP1) and CD163 (Cell Marque, Clone MRQ-26) was performed to visualize macrophages of type M1 and M2, respectively. The numbers of CD68-, CD163-, and Ki-67-positive cells were calculated per field of vision at high power magnification by two independent observers. M2 macrophages polarization was evaluated by calculating the CD163/CD68 ratio.

##### Statistics

One-way ANOVA multiple comparisons with the Tukey’s post-hoc analysis was used to assess the difference between groups using GraphPad Prism 8.0 software (V. 8.0.2, GraphPad Software, San Diego, CA, USA). *p* values of <0.05 were considered statistically significant.

## 3. Results and Discussion

### 3.1. AgNAs Characterization

**Figure 1:**
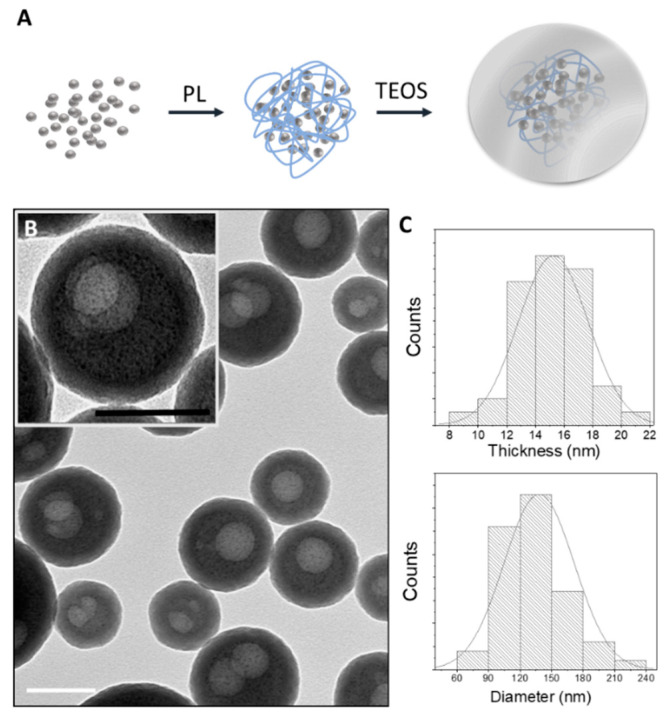
(**A**) General scheme of the formation of AgNAs. Silver nanoparticles and poly(l-lysine) (PL) self-arrange in controlled aggregates. Silica shells were grown on their surface due to hydrolysis of tetraethyl orthosilicate. (**B**) A wide-area TEM image of an AgNAs sample with a zoom in the inset. Scale bars: 100 nm. (**C**) Thickness distribution of shells (upper) and size distribution of NAs (bottom) plotted based on at least 100 particles imaged by TEM.

AgNAs are biodegradable silica nanocapsules of diameters of around 120 nm, comprising ultrasmall silver nanoparticles with sizes of about 1 nm. AgNAs production protocol as well as the characterization cascade assays are standardized [15,16]. The entire protocol requires less than 5 h for the production of 15 mg of AgNAs from a single operator, and the esteemed value of the nanoplatforms is about 1 EUR/mg when considering only the raw materials. After purification, particles were characterized via transmission electron microscopy in order to assess the passion fruit-like structure and to estimate the distribution of sizes and shell thicknesses (Figure 1). The silver content of AgNAs, evaluated by ICP-MS, was 3% w/w. AgNAs are produced and stored (maximally 1 year) at RT and, because they are maintained in 96% ethanol until their use, the sterility is ensured. Passion fruit-like nano-architectures (bio)degradation to clearable building blocks has been observed in a number of biological environments in less than 48 h, including full human serum, human blood, and cell cultures [20,21]. Noticeably, AgNAs can exploit the antimicrobial properties of silver nanoparticles avoiding the issue of metal persistence due to the clearance rate of the building blocks [15].

### 3.2. AgNAs In Vitro Antibacterial Study

Silver nanoarchitectures demonstrated a significant antibacterial activity with MIC of 10 µg/mL for *E. coli* and *P. aeruginosa*, and of 20 µg/mL for *S. aureus.*

**Figure 2:**
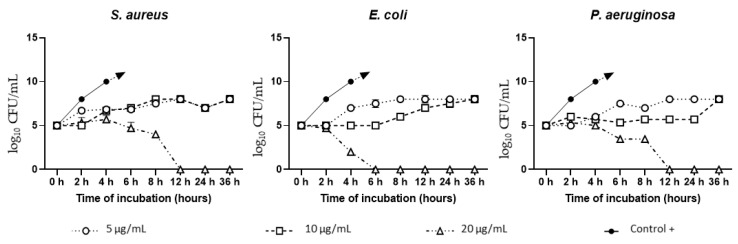
The time-dependent growth curves of bacteria after co-cultivation with different concentrations of AgNAs. In the positive controls (Control +) of all three bacterial species, log_10_ CFU/mL was >8 since the 4 h time point.

The time-dependent test showed that 20 µg/mL of AgNAs demonstrated significant antibacterial activity against all tested strains (Figure 2). Consequently, the bactericidal effect appeared after 6 h of co-cultivation with *E. coli* and after 12 h with *S. aureus* and *P. aeruginosa*. The antimicrobial behavior of AgNAs at the concentration of 10 µg/mL was more noticeable in the case of Gram-negative bacteria (*E. coli* and *P. aeruginosa*) than of the Gram-positive *S. aureus*. However, during the incubation, the possible Ag ions release provided bacteriostatic action differently, inhibiting bacterial growth in this concentration. All tested strains revealed minor sensitivity to silver nano-architectures in the concentration of 5 µg/mL. Although the AgNAs in this amount inhibited bacterial growth, at the final time point, the log_10_ CFU/mL reached the value of 8, similarly to the log_10_ CFU/mL of bacteria after co-cultivation with 10 µg/mL.

### 3.3. Cell Toxicity

**Figure 3:**
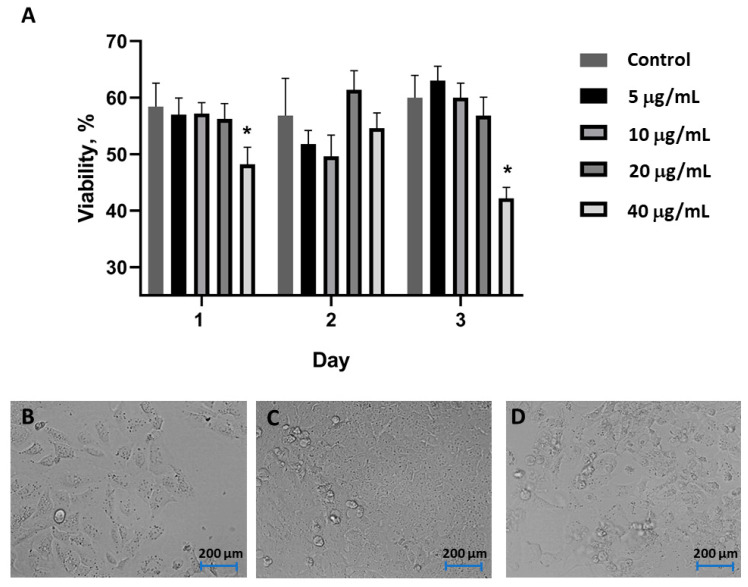
U2OS viability (**A**) evaluated by resazurin reduction assay for 3 days after the exposure to AgNAs at concentrations from 5 to 40 µg/mL. (**B–D**) Cell morphology assessment on days 1, 2, and 3 after exposure to 20 μg/mL AgNAs. *—significant differences between non-treated controls and AgNAs groups (*p* ≤ 0.05).

In agreement with standard protocols, the cell toxicity assessments were performed using an AgNAs concentration of over twice the MIC. Concentrations of AgNAs comprised between 5 and 20 µg/mL did not affect U2OS cells within 3 days. Indeed, cells demonstrated a proliferation profile similar to the one of the control group. On the other hand, a significant reduction in cell activities was observed on days 1 and 3 after exposure to AgNAs at concentration of 40 µg/mL (Figure 3A). U2OS cell growth and proliferation from day 1 to day 3 (under exposure to 20 µg/mL of AgNAs) was confirmed by optical microscopy (Figure 3C–D). Indeed, a majority of cells had the typical morphology and proliferation activity. The cytoplasm of some cells contained grain-like structures, which could be probably associated with the internalization of AgNAs. Overall, the actual cellular biosafety profile of AgNAs taken together with the whole-body toxicity profiles already investigated on vertebrate models [15] and the MIC recorded for *E. coli*, *S. aureus*, and *P. aeruginosa* confirmed the potentiality of silver nano-architectures for in vivo investigations on wound healing.

### 3.4. Animal Experiment

**Figure 4:**
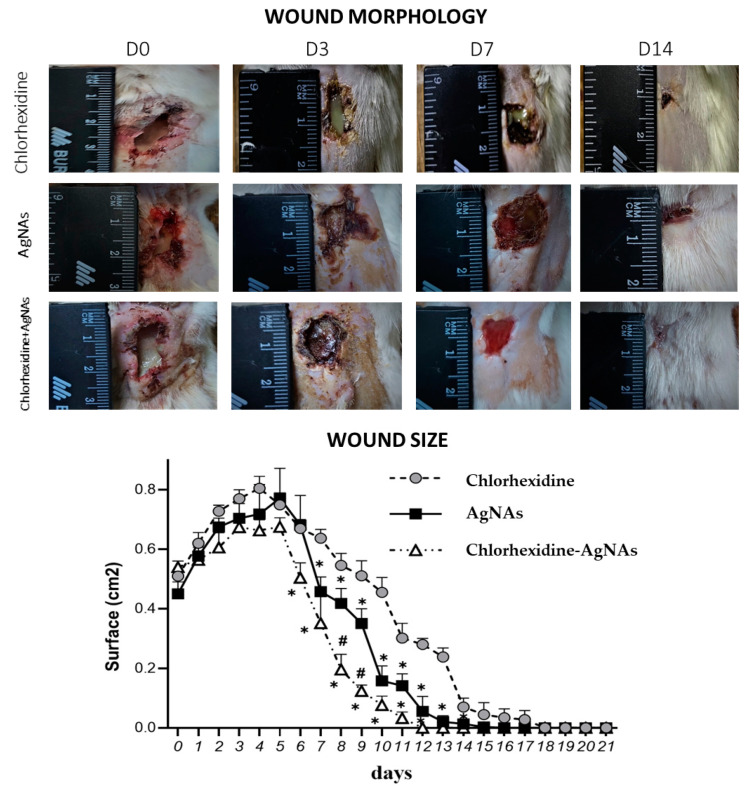
Upper section—photographs of the wound at different time-points after the application of the treatments (chlorhexidine, AgNAs, and combined chlorhexidine–AgNAs). On day 21, all the experimental groups experienced a complete wound epithelization. Lower section—wound size measurements after the treatment by chlorhexidine, AgNAs, and combined chlorhexidine–AgNAs. *—significant difference from the chlorhexidine group (*p* ≤ 0.05); #—significant difference between AgNAs and chlorhexidine–AgNAs groups (*p* ≤ 0.05).

The evaluation in wound healing progress was quantified by measuring the square of wounds treated with different formulations (chlorhexidine—Ch, AgNAs, and combined AgNAs–chlorhexidine). In order to comprehensively assess the efficacy of AgNAs, the actual standard of care for wound healing (treatment with chlorhexidine) was employed as control. Figure 4 demonstrates wound healing dynamic from day 0 to day 14. Both antibacterial AgNAs per se and in combination with chlorhexidine led to a significant decrease in the purulent process from day 3, compared with the chlorhexidine group (day 9). Noticeably, purulent detritus was still present in chlorhexidine-treated animals on day 7, in contrast with the clean wound in the experimental groups treated with AgNAs (Figure 4). The signs of clearance from pathological detritus in AgNAs and chlorhexidine–Ag groups appeared from day 5–6, while in the chlorhexidine-treated animals, it was from day 9. Remarkably, the wound closure process was significantly facilitated from day 7 in chlorhexidine–Ag group and the wound was completely closed on day 14, while it happened on days 16 and 18 for AgNAs and chlorhexidine groups, respectively. It should be noted that at the last time-point (day 21), the wounds in all groups were closed with epithelization.

As expected, the wound size slightly increased up to day 4 in all groups due to purulent inflammatory reaction, with following contraction from day 5 (Figure 4). Due to the antibacterial and anti-inflammatory features of silver, a significant contraction in the chlorhexidine–AgNAs group was observed, in comparison with the pure chlorhexidine treatment, on day 6. A significant wound size reduction in the group treated with AgNAs alone appeared on day 7. The wound size significantly differed between chlorhexidine control and both AgNAs groups at all time-points until day 16, while chlorhexidine–AgNAs demonstrated a significant difference from pure AgNAs on days 8 and 9 (Figure 4, lower section).

**Figure 5:**
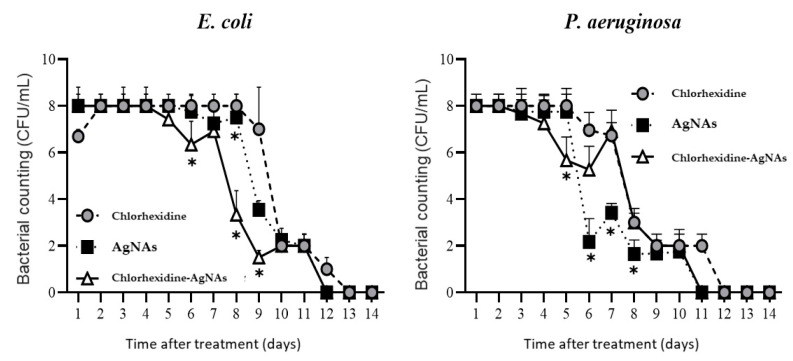
Wound microbiological profile at different times after treatment with chlorhexidine, chlorhexidine–AgNAs, and AgNAs solutions. *—significant difference from the control (chlorhexidine) groups (*p* ≤ 0.05).

The bacteriological profile of the purulent wound suggested that conventional chlorhexidine treatment leads to reduction in *E. coli* and *P. aeruginosa* from days 9 and 6, respectively (Figure 5). It should be noted that *S. aureus* has not been determined due to competitive inhibition between the bacteria during the wound modelling [20]. AgNAs alone and in combination with chlorhexidine provided a strong antibacterial effect that led to bacteria reduction in purulent wounds from day 5 for *P. aeruginosa* and from day 6 for *E. coli.* Unexpectedly, pure AgNAs provided a significantly better wound clearness from *P. aeruginosa* than the AgNAs–chlorhexidine group, while negligible differences were observed for AgNAs groups in *E. coli* eradication from purulent wound. Remarkably, bacterial persistence is the main factor that influences wound healing, and a significant reduction of microorganisms penetration can be a crucial factor in order to promote skin regeneration [2].

### 3.5. Histological Evaluation of Wound Healing

#### 3.5.1. AgNAs Accelerated Wound Cleansing and Shortened the Inflammatory Phase of the Wound Process

**Figure 6:**
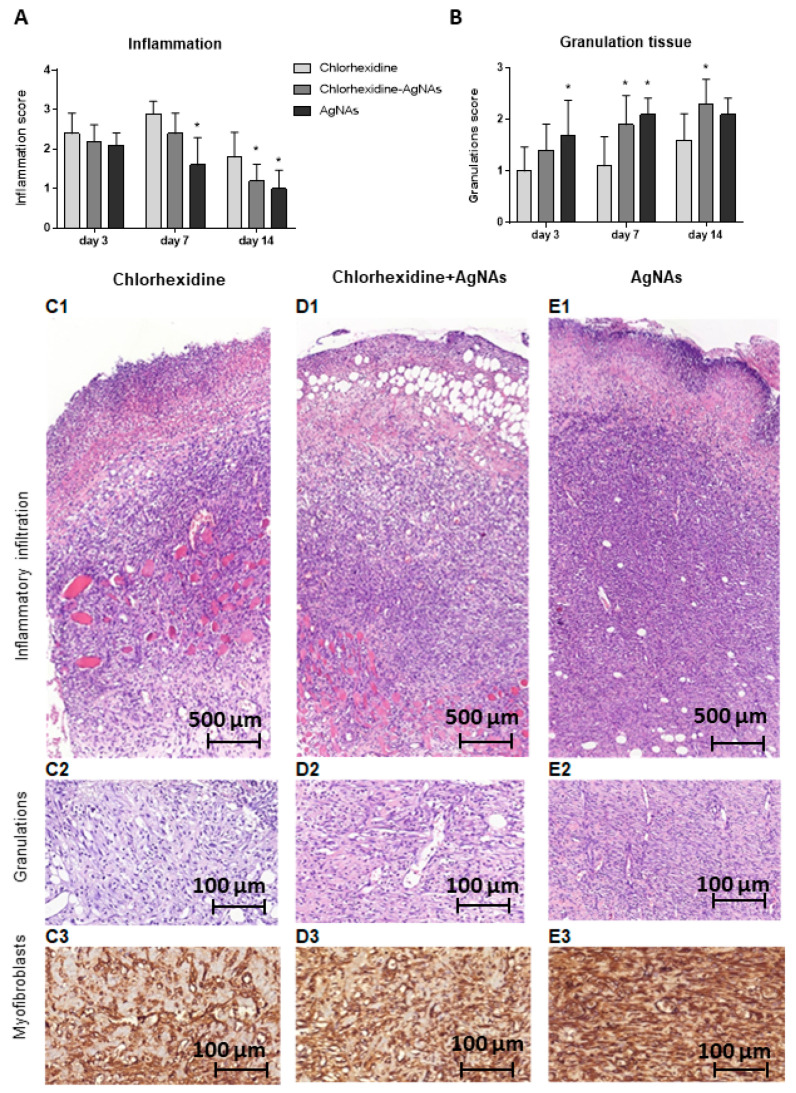
Inflammatory infiltration (**A**) and granulation (**B**) tissue formation in wounds treated with chlorhexidine (**C**), chlorhexidine–AgNAs (**D**) or AgNAs (**E**). The layer of granulation tissue was considerably thicker in rats treated with AgNAs (**E**) or chlorhexidine–AgNAs (**D**) compared with dressing with chlorhexidine. **C1,2; D1,2;** and **E1,2**: Staining with hematoxylin and eosin. **C3–E3**: Immunohistochemistry to alpha-smooth muscle actin (aSMA). *—significant difference from the control (chlorhexidine) groups (*p* < 0.05).

Histologically, AgNAs-application was associated with reduction in both secondary tissue damage and inflammation at the early stages of treatment. On day 3, wound beds were covered by purulent detritus with microbial colonies and cellular debris. Numerous polymorphonuclear leukocytes (PMN) infiltrated soft tissues (fat and muscles) at wound bottom and margins. Although on day 3, the inflammatory reaction did not differ significantly between groups, on day 7, the density of PMN-infiltration was significantly lower in wounds treated with AgNAs (*p* = 0.017) when compared with the Ch-group. Notably, at this time, Ch-dressed wounds demonstrated increased inflammatory score due to progressive tissue damage. In contrast, AgNAs-treated rats had localized injury with lower PMN infiltration and edema (Figure 6). After 2 weeks of treatment, all wound showed reduced PMN-infiltration. However, the lowest inflammatory score was found for AgNAs-treated rats (*p* = 0.022), while Ch-dressed wounds showed features of persistent inflammation.

#### 3.5.2. AgNAs Application Facilitates Wound Healing

As a result of inflammatory phase control, the AgNAs-treated group demonstrated a significantly higher rate of granulation tissue formation. The most significant differences in the pace of repair between groups have been found on day 7 (*p* < 0.001). In particular, both AgNAs and Ch-AgNAs dressed wounds demonstrated accelerated granulation tissue formation relative to the control.

Comparing the dynamic of granulation tissue growth in rats with either AgNAs application or chlorhexidine treatment, we found significant differences in the thickness of granulations and density of aSMA-positive (alpha-smooth muscle actin) myofibroblasts. These cells are pivotal for granulation tissue, essential for cell-matrix mechanical interplay and wound contraction. Additionally, they produce a wide range of growth factors (including FGF, VEGF, EGF, and NGF) promoting angiogenesis, keratinocytes proliferation, and wound epithelization [22,23]. Furthermore, the arrangement of the newly formed vessel differed between the groups. While Ch-dressed wounds demonstrated mostly irregular patterns of widened vasculature, the granulations of AgNAs-treated rats showed numerous thin, parallelly arranged vascular profiles. These data reflect the accelerated switch from the inflammatory phase to granulation tissue formation and angiogenesis under AgNAs-treatment. Several possible mechanisms can explain the beneficial effects of AgNAs. For example, AgNAs provide a prominent antimicrobial effect facilitating the purulent wound cleansing. Moreover, AgNAs prevent the expanded secondary damage caused by conventional antiseptic agents due to a direct toxic effect on host cells. Indeed, although chlorhexidine gluconate is widely used for surgical skin preparation and as an intra-wound irrigation agent with significant antimicrobial effects, its application at clinical concentrations results in a significant cytotoxic effect. In vitro investigations have demonstrated that chlorhexidine permanently inhibited cell migration and significantly reduced the survival of fibroblasts, myoblasts, and osteoblasts [24]. Additionally, the application of chlorhexidine inhibits the fibroblasts proliferation and their secretory activity, reducing the levels of collagens and non-collagen proteins [25]. In contrast, AgNAs have not demonstrated significant cytotoxic effects. Furthermore, silver-containing nanomaterials usually enhance cell adhesion, migration, proliferation, ROS scavenging, and de novo tissue formation [26,27,28]. This evidence may explain the differences in granulation tissue formation and wound healing between the different groups considered in this investigation. It is worth to notice that AgNAs have also demonstrated an interesting stimulating effect on the dermis repair.

#### 3.5.3. AgNAs Impact Macrophages Polarization Promoting Wound Phases Progress

Myofibroblasts formation depends on various factors, among which the transforming growth factor β (TGFβ), which is an essential inducer of myofibroblast differentiation derived from macrophages [29]. These cells are polymorphous and can participate in various processes during wound healing [30]. Initially, macrophages produce a broad range of proinflammatory cytokines and chemokines enhancing the inflammatory phase with PMN-leukocytes activation and recruitment [31]. Moreover, macrophages are responsible for phagocytosis of apoptotic neutrophils (or efferocytosis) [32]. This event can induce macrophages transition from the proinflammatory (M1) to the anti-inflammatory (M2) phenotype [33]. M2 type macrophages reduce the inflammation and stimulate tissue repair by generating anti-inflammatory cytokines and growth factors, such as transforming growth factor-β (TGF-β) and VEGF [34]. These growth factors are essential for angiogenesis, myofibroblast proliferation, and extracellular matrix synthesis [35].

**Figure 7:**
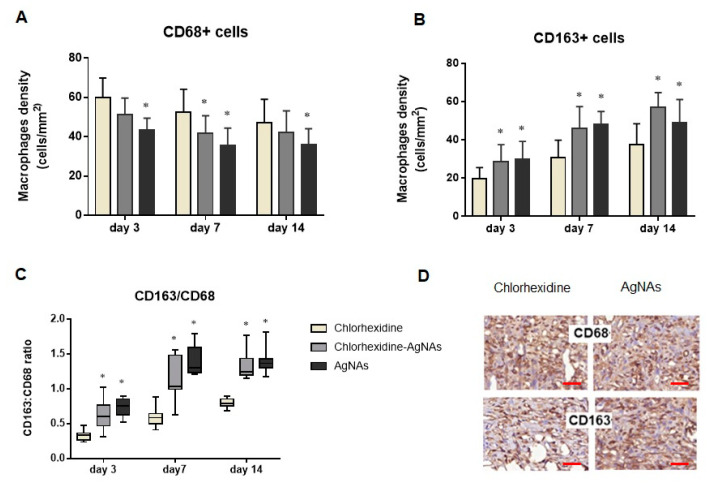
Number of M1 and M2 macrophages during wound healing in rats with different treatments of wounds. The graphs in (**A**) and (**B**) demonstrate changes in the number of CD68+ and CD163+ cells, respectively; the graph in (**C**) indicates a comparison of the CD163/CD68 ratios reflecting M2 macrophages polarization; photographs in (**D**) (scale bar—100 µm) illustrate differences in various macrophage numbers in the group treated with chlorhexidine compared with AgNAs application. *—significant difference from the control (chlorhexidine) groups (*p* ≤ 0.05).

To clarify the possible mechanisms of AgNAs effects on wound healing, we assessed macrophages as the key cells regulating inflammation and tissue repair. The distribution and number of M1 (CD68+) and M2 macrophages (CD163) were analyzed. Interestingly, from day 3 after treatment, the quantity of CD68+ cells was higher in chlorhexidine-treated rats (*p* < 0.001) compared with the other groups. In contrast, AgNAs application was associated with higher CD163+ cells number (*p* = 0.003) over the whole period of the investigation (Figure 7). It worth noting that the AgNAs application alone or in combination with chlorhexidine resulted in an increased CD163/CD68 ratio that reflected a shift in macrophages toward the M2 phenotype, i.e., an accelerating granulation tissue formation and inflammation reduction.

This finding is of special interest as M1 to M2 transition is essential for resolving inflammation and for an effective repair [36,37]. Enhanced M2 polarization under AgNAs application is in agreement with our findings regarding the terms of inflammation resolution and granulation tissue performance. Under pathological conditions, M2 macrophages activation is induced by fungi, parasites, immune complexes, complements, apoptotic cells, macrophage colony-stimulating factor (MCSF), interleukin-4 (IL-4), IL-13, IL-10, and various other signals [38]. Recent studies have shown that the application of Ag-containing nanomaterials can modulate macrophages polarization in vitro and in vivo by facilitating the M2 phenotype transition. This effect was due to cell signaling effects by regulating the glucose transporter GLUT1 and autophagy mechanisms [39]. Similarly, AgNAs facilitate M2 macrophages during purulent wound healing. However, in order to finely shed light on the molecular mechanisms of AgNAs immunomodulating effects, further investigations are needed.

## 4. Conclusions

Overall, AgNAs demonstrated interesting antimicrobial and anti-inflammatory behaviors and, in particular, a (i) stimulating effect on wound healing, (ii) spatial and temporal reduction in purulent inflammation, and (iii) acceleration of the proliferation phase by assisting the granulation tissue formation and wound epithelization. The histological analysis and the macrophage quantification confirmed that the wound healing promotion effect of AgNAs is associated with the enhancement of M2 macrophages polarization. The antimicrobial and anti-inflammatory performances of AgNAs, taken together with their geometry and intrinsic non-persistence, pave the way for the composition of multifunctional nanoplatforms for the efficient topical (co)treatment of surgical site infections. Moreover, due to the AgNAs structure and resistance to nebulization, they can be further investigated for the treatment of pulmonary infections.

## Data Availability

The data presented in this study are available on request from the corresponding authors.
